# Suicide in Hungary-epidemiological and clinical perspectives

**DOI:** 10.1186/1744-859X-12-21

**Published:** 2013-06-26

**Authors:** Zoltan Rihmer, Xenia Gonda, Balazs Kapitany, Peter Dome

**Affiliations:** 1 Department of Clinical and Theoretical Mental Health, Faculty of Medicine, Semmelweis University, Budapest, Hungary; 2 Department of Psychiatry and Psychotherapy, Faculty of Medicine, Semmelweis University, Budapest, Hungary; 3 Demographic Research Institute of the Hungarian Central Statistical Office, Budapest, Hungary

**Keywords:** Hungary, Suicide, Suicidal behavior, Epidemiology, Risk factors, Prevention

## Abstract

Annual suicide rates of Hungary were unexpectedly high in the previous century. In our narrative review, we try to depict, with presentation of the raw data, the main descriptive epidemiological features of the Hungarian suicide scene of the past decades. Accordingly, we present the annual suicide rates of the period mentioned and also data on how they varied by gender, age, urban vs. rural living, seasons, marital status, etc. Furthermore, the overview of trends of other factors that may have influenced suicidal behavior (e.g., alcohol and tobacco consumption, antidepressant prescription, unemployment rate) in the past decades is appended as well. Based on raw data and also on results of the relevant papers of Hungarian suicidology we tried to explain the observable trends of the Hungarian suicide rate. Eventually, we discuss the results, the possibilities, and the future tasks of suicide prevention in Hungary.

## Introduction

Suicidal behavior is a major public health problem worldwide, and its prediction and prevention receives increasing attention. Approximately 1 million people die by suicide in the world every year, and it is estimated that 1.5 million will die from suicide in 2020. The global suicide rate of the world is 14 suicides per 100,000 inhabitants: more specifically, 18 suicides per 100,000 males and 11 suicides per 100,000 females [1,2]. In spite of the fact that suicide rates of different countries and continents differ substantially, the rate of completed suicide, in general, is much higher among males, among older people, and among Caucasians.

Although suicide is a very complex, multi-causal behavior, involving several medical-biologic and psycho-social components, history of untreated major psychiatric (particularly depressive and alcohol-related) disorders constitute the most important risk factors. However, lifetime and current psycho-social and personality factors (stressful life events, financial problems, unemployment, impulsivity) and other forms of addictive behaviors than drinking (e.g., cigarette smoking) have been also found to be in a statistically significant positive relationship with suicide mortality [3-8].

The highest annual suicide rates are reported from Eastern Europe (13–42 suicides per 100,000 persons) followed by Western/Nordic European countries (8–21 suicides per 100,000 persons) and North America and Australia (11–13 suicides per 100,000 persons). Latin America, as well as ‘Latin Europe’ (Greece, Spain, Italy), and Central Asian countries report annual suicide rates less than 10 [1,9,10]. Reasons for these great differences between national/regional suicide rates have not been fully explained. Geographic, climatic, socio-cultural, dietary, religious, and economic differences can be taken into account, but differences in the psychiatric morbidity as well as the accuracy of the registration of suicide, the stigma associated with suicide possibly influencing reporting rates, the availability of lethal methods, and the availability of the social/health care system should also be considered [4,11].

The primary aim of our narrative review, based on both raw data retrieved from the databases of the Hungarian Central Statistical Office and papers published in international journals (identified by Pubmed searches using keyword combinations ‘suicide + Hungary’, ‘suicidal + Hungary’; ‘suicide + Hungarian’; ‘suicidal + Hungarian’) and also on papers/books in Hungarian (identified by Google), is to present the main characteristic features of the Hungarian suicide scene of the last 50 years and also to append some possible explanations of the trends observed.

## Review

### Suicide in Hungary-an introduction

Suicide rates are high in large parts of Northern and Eastern Europe, and some of the highest figures have been reported in Hungary [1,10]. In addition to psycho-social factors, several lines of evidence indicate genetic and biological contributions to unexpectedly high Hungarian suicide rate [12]. Within Europe, the countries with the highest suicide rate constitute a contiguous J-shaped belt from Finland through the Baltic countries, Russia, Belarus, and Ukraine to central Europe (Hungary, Slovenia, Austria) [12]. Genetic similarities observed between populations of these countries led to the Finno-Ugrian suicide hypothesis which states that high suicide rates of these countries are the consequence of a shared genetic susceptibility [12]. Genetic background of this phenomenon is very probable because other (e.g., cultural/socio-political/economic) features of these countries are quite different. Consonant with the theory about the genetic background of the high suicide rate of Hungary, Hungarian immigrants in the USA have the highest suicide rates of all immigrant groups [11]. In addition, the existence of an unfortunate genetic/cultural susceptibility of Hungarians to suicidal behavior is further bolstered by the fact that suicide rates of those Romanian counties where the proportions of Hungarian people are high (e.g., Harghita, Covasna, Mures) were much higher than of those counties where the population percentages of Hungarians are low [13].

Between 1960 and 2000 in the vast majority of years, the suicide rate of Hungary was the highest in the world. The reason of this very high suicide mortality of Hungary is not fully understood. It is one possibility is that the medical examiners in Hungary certify those deaths as suicide which would otherwise be labeled as undetermined death or as death related to other causes. However, the highest suicide rate of Hungarian immigrants in the USA [11] and the similarly high suicide rate of ethnic Hungarians living in Romania [13] contradict this possibility. Political or economic causes are also very unlikely, as between 1960 and 1990 the suicide rates of Poland, Bulgaria, Romania, and former Yugoslavia (countries with similar political and economic systems) were around one third of the Hungarian figure, and during the mentioned period, in the majority of the years, the suicide mortality of Denmark, Finland, Austria, and Sweden (with much more advantageous political and economic situations) have been among the top ten in the world. As mentioned above, the most established risk factor(s) of suicide are different forms of (untreated) major affective disorders. Although direct comparison of national epidemiological data on prevalences of affective disorders is not fortunate due to some methodological issues (e.g., different studies have frequently used different diagnostic instruments), it can be said that lifetime prevalence of ‘any’ bipolar disorder, which carries the highest risk of suicide [14-16], is unusually high in Hungary (5.1%) [17,18]. Albeit, lifetime prevalence of major depressive disorder according to the DSM-IV criteria in the Hungarian population (15.1%) is similar to the corresponding data from other European countries and the USA, a recent study, assessing depressive symptoms using CES-D in the general population of 23 European countries, reported the highest mean scores in Hungary among all investigated countries [19,20]. In summary, these results raise the possibility that high prevalence of affective (especially bipolar) disorders (and possibly also subthreshold manifestations of bipolarity and bipolar spectrum disorders) in the Hungarian population may be one of the most important contributors to the markedly high suicide rate of Hungary.

Looking at European countries with the highest suicide rates 25 years ago (between 20 and 46 per 100,000 per year), the 26%–54% decline in the national suicide rates of Hungary, Denmark, Germany, Austria, Estonia, Switzerland, Sweden, and Finland in the last 2 decades is quite impressive [3,10,21-23]. However, given the recent economic crisis, suicide rates are stabilized or show a slight increase in many European countries [24,25].

### Suicide mortality of Hungary between 1961 and 2010

#### Time trends, gender distribution, and method of suicide

**Figure 1:**
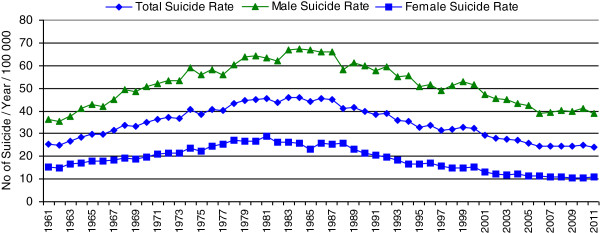
Total, male, and female suicide rates (per 100,000/year) in Hungary from 1961 to 2011.

Figure 1 and Table 1 show the suicide rate of Hungary between 1961 and 2011. There was a steady increase in suicide mortality between 1961 (25.5/100,000/year) and 1983. The peak of suicide rate was in 1983 (45.9/100,000/year). After 1983, the suicide rate remained virtually unchanged (≈44–45/100,000/year) until the year 1988 when a sharp and longstanding decline began (this steady decline lasted until 2007 (24.3/100,000/year), and the suicide rate stabilized at this level up to now). By virtue of the striking coincidence of the beginning of decline in (total) suicide rate and the political transition in Hungary (1988–1989) some authors raised the possibility that the termination of the communist regime was reflected in the decrease of suicide rate. At the same time, however, the increasing trend of female suicide rate turned into a decline already in the beginning of the 1980s so the beneficial effect of political transition on suicide rate, at least in the case of females, is questionable [26].

**Table 1:** **Suicide rates (*n*/100,000/year) for Hungarian total, male, and female populations between 1961 and 2011**

**Year**	**Total suicide rate**	**Male suicide rate**	**Female suicide rate**
1961	25.50	36.41	15.33
1962	24.90	35.60	14.91
1963	26.80	37.53	16.78
1964	28.60	40.94	17.08
1965	29.89	42.68	17.92
1966	29.63	42.10	17.98
1967	31.38	45.18	18.47
1968	33.80	49.23	19.35
1969	33.20	48.41	18.94
1970	34.84	50.76	19.86
1971	36.09	52.06	21.07
1972	36.99	53.49	21.46
1973	36.92	53.24	21.55
1974	40.79	59.03	23.62
1975	38.56	55.95	22.17
1976	40.74	58.10	24.38
1977	40.39	56.13	25.56
1978	43.20	60.51	26.89
1979	44.58	63.64	26.62
1980	44.90	64.45	26.54
1981	45.55	63.47	28.73
1982	43.50	61.99	26.15
1983	45.90	66.74	26.36
1984	45.89	67.51	25.64
1985	44.34	66.94	23.20
1986	45.27	66.04	25.88
1987	45.02	65.87	25.57
1988	41.28	58.09	25.60
1989	41.52	61.33	23.06
1990	39.84	59.78	21.39
1991	38.56	57.92	20.68
1992	38.70	59.25	19.73
1993	35.83	54.92	18.24
1994	35.27	55.39	16.77
1995	32.88	50.53	16.68
1996	33.67	51.64	17.19
1997	31.59	49.10	15.55
1998	32.04	50.99	14.70
1999	32.98	52.93	14.75
2000	32.55	51.40	15.35
2001	29.21	47.04	13.03
2002	27.94	45.38	12.14
2003	27.62	44.85	12.02
2004	27.10	43.44	12.33
2005	25.96	42.31	11.18
2006	24.42	38.90	11.34
2007	24.34	39.32	10.80
2008	24.66	40.07	10.73
2009	24.53	39.93	10.61
2010	24.88	40.89	10.40
2011	24.25	38.93	10.97

Source: Hungarian Central Statistical Office, Budapest, Hungary.

In Hungary, similar to the great majority of developed countries, males consistently show much higher suicide rates than females [2,27]. However, the female suicide rate decreased more markedly between 1983 and 2010 (females, 61% decline; males, 39% decline) and while the male-to-female suicide ratio was 2.53 in 1983, the same figure was 3.93 in 2010.

Table 2 displays the distribution of violent and nonviolent suicide cases by gender in the years 1980, 1995, and 2010. In accordance with world trends, the proportion of violent suicides among all suicides is higher in males than in females in Hungary [27,28]. Although during this 30-year period, the total suicide mortality of Hungary has decreased substantially, the proportion of violent suicide cases has increased markedly and strictly both in males and females (Table 2). This can reflect the fact that improved care of psychiatric patients (including less toxic psychotropics and higher level of intensive care) can save more lives in the case of nonviolent attempts (overdose, poisoning), while violent suicide methods (hanging, shooting, drowning, etc.) are more rapid and mostly irreversible [29].

**Table 2:** Distribution (%) of violent and nonviolent suicides in Hungarian populations in 1980, 1995, and 2010

**Population**	**Method of suicide**	**1980**	**1995**	**2010**
Male	Violent	77.4%	85.81%	89.9%
	Nonviolent	22.6%	14.19%	10.1%
Female	Violent	51.5%	59.02%	67.3%
	Nonviolent	48.5%	40.98%	32.7%

Source: Hungarian Central Statistical Office, Budapest, Hungary.

In line with data from the majority of other countries, suicide attempts are more prevalent among females than among males in Hungary as it was suggested by results of studies conducted in consecutive series of suicide attempters in different areas of the country (Budapest, Pécs, Győr) and also in a sample representative of the Hungarian population (n = 2.953; number of suicide attempters = 94) [15,30-32]. According to the unpublished data of the Hungarian National Ambulance Emergency Service, the number of ‘suicide events’ (which mainly mirrors suicide attempts) decreased in a monotonic and relevant manner from 1986 to 2006 (from 23,729 to 8,025). After 2007 (till 2011), the values were between 8,256 and 8,795 (Göbl and Andics, personal communication). Accordingly, trends in suicide attempts roughly followed the trends in completed suicide (a decrease from the mid 1980s to 2006 and a stabilization period from 2007 till now). Previously, we have already provided some supposed explanations (and later we will provide others) for the decreasing part of the curve of suicide rate (observable from the mid 1980s to 2006). However, there are no exact explications to the disappearance of the declining trend from the suicide rate from 2007 till now, it is probable that increasing unemployment and restrictions/destructions with which Hungarian psychiatry had to be faced in the last few years may contribute to this unfavorable turn [25,33].

#### Alcohol consumption, antidepressant prescription, unemployment, and cigarette smoking

Table 3 shows the data on alcohol consumption, antidepressant prescription, unemployment, and tobacco consumption in Hungary between 1961 and 2011. All of them have been reported in the literature to be in a statistically significant relationship with suicide mortality; unemployment, alcohol, and tobacco consumption shows a positive, and antidepressant prescriptions a negative correlation [3,5-8,23,34,35]. However, during the time period from 1985 to 2008, only antidepressant prescription and tobacco consumption showed significant associations (a negative and a positive, respectively) with suicide mortality in Hungary while alcohol consumption and changes in GDP did not [8].

**Table 3:** Alcohol and tobacco consumption, antidepressant use, unemployment rates and total suicide rates in Hungary

**Year**	**Pure (absolute) alcohol consumption (in liters per capita)**	**Antidepressant use (DDD/1,000 inhabitants/day)**	**Unemployment rate (total)**	**Unemployment rate (male)**	**Unemployment rate (female)**	**Tobacco consumption (in kilogram per capita)**	**Total suicide rate (per 100,000/year)**
1961	6.1						25.50
1962	6.2						24.90
1963	6.5						26.80
1964	7.3						28.60
1965	6.8						29.89
1966	6.9						29.63
1967	7.5						31.38
1968	7.7						33.80
1969	8.7						33.20
1970	9.1						34.84
1971	9.5						36.09
1972	9.5						36.99
1973	9.5						36.92
1974	9.4						40.79
1975	10.1						38.56
1976	10.7						40.74
1977	11.3						40.39
1978	11.5						43.20
1979	11.1						44.58
1980	11.7						44.90
1981	11.5						45.55
1982	11.6						43.50
1983	11.4						45.90
1984	11.7						45.89
1985	11.6	2.67				2.1	44.34
1986	11.5	2.74				2.2	45.27
1987	10.8	2.80				2.1	45.02
1988	10.5	3.10				2.1	41.28
1989	11.3	3.40				2.2	41.52
1990	11.1	3.70				2.0	39.84
1991	10.7	3.80				1.8	38.56
1992	10.5	3.90	9.9	11.0	8.7	1.7	38.70
1993	10.6	4.03	12.1	13.5	10.4	1.6	35.83
1994	10.5	5.18	10.8	12.1	9.4	1.6	35.27
1995	10.0	6.48	10.3	11.6	8.7	1.5	32.88
1996	10.3	8.13	10.0	10.9	8.8	1.4	33.67
1997	10.1	10.13	8.8	9.6	7.8	1.5	31.59
1998	10.2	12.29	7.8	8.5	7.0	1.6	32.04
1999	10.1	13.28	7.0	7.5	6.3	1.7	32.98
2000	10.0	13.65	6.4	7.0	5.6	1.5	32.55
2001	11.1	16.28	5.7	6.3	5.0	1.5	29.21
2002	11.2	18.28	5.8	6.1	5.4	1.5	27.94
2003	11.1	21.25	5.9	6.1	5.6	1.5	27.62
2004	11.1	20.88	6.1	6.1	6.1	1.4	27.10
2005	11.0	23.57	7.2	7.0	7.5	1.4	25.96
2006	11.2	25.41	7.5	7.2	7.8	1.5	24.42
2007	10.7	24.61	7.4	7.1	7.6	1.5	24.34
2008	10.0	26.24	7.8	7.6	8.1	1.6	24.66
2009	9.8	26.33	10.0	10.3	9.7		24.53
2010		27.08	11.2	11.6	10.7		24.88
2011		27.60	10.9	11.0	10.9		24.25

Data taken between 1961 and 2011. Source: Hungarian Central Statistical Office, Budapest, Hungary; [8,25].

#### Age distribution, marital status

Table 4 shows the age distribution of suicide cases in the total, male, and female populations. In Hungary, similarly to the majority of European countries, total, male, and female suicide rates mainly increase with age (there are also some exceptions, e.g., Ireland (in both genders) and Finland (in females), whose suicide rate reaches its maximum in middle-aged cohorts and declines in elderly) [2,36]. A plateau (or a slight decrease) of the suicide rate after midlife (in cohorts aged ≈ 60–70 years old) was also observable in the case of the Hungarian total population in the year 2010. This bimodal pattern (with a smaller maximum of the curve in the cohorts aged 30–50 (60) years and a bigger maximum of the curve after age 75) is slightly reminiscent of findings from some other European countries (e.g., Austria, Belgium, Czech Republic, Estonia, Latvia) [2].

**Table 4:** **Distribution of suicide rates (*n*/100,000/year) by age in populations in Hungary in 1980 and 2010**

**Age group (years)**	**Total population**		**Male population**		**Female population**	
	**SR (1980)**	**SR (2010)**	**SR (1980)**	**SR (2010)**	**SR (1980)**	**SR (2010)**
10–19	8.2	3.4	11.90	4.58	4.27	2.22
20–29	30.3	11.8	48.42	19.16	11.57	4.09
30–39	48.6	19.1	74.88	32.04	22.13	5.59
40–49	63.0	35.1	94.79	57.28	33.34	13.23
50–59	68.1	42.5	104.53	72.39	36.21	16.11
60–69	72.1	37.8	98.64	65.18	51.20	16.95
70–79	102.0	39.9	157.20	79.22	64.62	17.25
80–89	138.6	49.7	211.81	114.53	101.84	21.83
90 and above	192.6	70.3	337.66	146.20	134.74	41.52

SR, suicide rate. Source: Hungarian Central Statistical Office, Budapest, Hungary.

Table 5 shows suicide rates in groups with different marital statuses. As it can be seen, the highest suicides rates are associated with divorced and widowed statuses while the lowest rates can be observed among those who are married. Investigating the regional distribution of divorce rates and suicide rates in 20 regions of Hungary we have previously reported a significant positive association; higher suicide rate in the given region was associated with higher divorce rate and vice versa [37]. These findings are in accordance with observations from other countries [36,38-40].

**Table 5:** **Hungarian suicide rates (*n*/100,000/year) in groups with different marital status in 1980 and 2010**

**Marital status**	**Gender**	**Age (years)**	**1980**	**2010**
Unmarried	Male	15–39	50.94	20.71
		40–59	145.52	80.14
		60 and above	131.27	145.32
	Female	15–39	13.97	5.38
		40–59	58.93	17.97
		60 and above	59.62	12.14
Married	Male	15-39	46.93	23.27
		40-59	84.7	43.31
		60 and above	103.35	52.18
	Female	15-39	12.35	2.94
		40-59	26.56	9.72
		60 and above	40.03	9.1
Widowed	Male	15-39	229.8	196.17
		40-59	233.8	208.96
		60 and above	272.7	155.38
	Female	15-39	65.84	0.0
		40-59	62.34	24.26
		60 and above	78.36	23.72
Divorced	Male	15-39	189.76	63.11
		40-59	251.88	117.16
		60 and above	172.37	123.21
	Female	15-39	47.75	7.44
		40-59	68.15	24.52
		60 and above	87.84	27.25

Source: Hungarian Central Statistical Office, Budapest, Hungary.

#### Regional distribution, urban–rural differences

**Figure 2:**
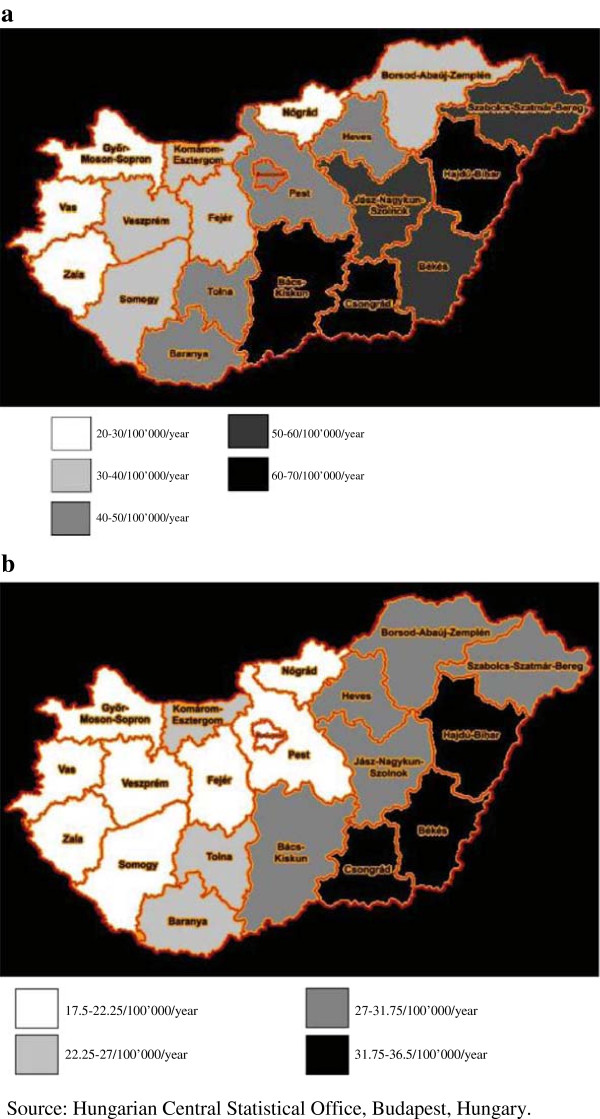
Suicide rates in Hungarian counties in the year (a) 1980 and (b) 2010.

Similarly to some other countries, marked regional differences in suicide rates are present in Hungary [41-43]. As shown in Figure 2a, b the suicide rates are higher in the south-eastern than in the north-western parts of the country. This pattern is quite stable in time (the first mention of this phenomenon was in the year 1864) [42-44]. Although some possible explanations of the spatial inequality of suicide rates have been proposed (e.g., the proportion of Protestants is higher in the south-eastern region; there are also differences in attitudes toward suicide and levels of social integration in the Durkheimian sense between populations with high vs. low suicide rates) the exact explanations are still missing [26,42,43]. The only study that tried to explore the cause(s) of the marked regional differences in suicide mortality in Hungary based on exact data found a significant negative correlation between the suicide rates and the rates of treated depressions in 19 counties of Hungary; the higher was the rate of recognized (treated) depression the lower was the suicide rate in the given county [42]. However, this cannot be a full explanation, because the mentioned regional difference in suicide mortality of Hungary has been present several decades before the introduction of antidepressants. However, looking at the severity of depressive symptoms, as measured by the Beck Depression Inventory, in a representative sample of Hungarian population it was found that the highest mean total Beck scores were found in North-East Hungary, where the suicide rates were also the highest [45,46]. Intriguingly, some results suggest that those subjects who were born in Hungarian areas with high-suicide rates have a higher chance of committing suicide after relocating to other regions from their place of birth [43,47]. It is also noteworthy that differences between suicide rates of districts in the capital city Budapest (some of them having a number of inhabitants greater than 100,000) are also remarkable. For example, in the year 1990, the district with the highest suicide mortality had a 49.1/100,000/year suicide rate, while the district with the lowest suicide mortality had a 20.5/100,000/year suicide rate in Budapest.

Table 6 shows the trends in suicide rates of the total, male, and female populations according to an ‘urban-rural’ approach. Although, as shown in the table, in 1970 there was no remarkable difference between the suicide rates of urban and rural areas in the total population, a growing gap between urban and rural suicide rates is observable on the long run. Accordingly, a clear trend has evolved up to 2010: the greater the level of urbanicity, the lower the level of suicide rate. This phenomenon is in accordance with observations from several other countries [48]. In females, there was an obvious association between domicile and suicide rate in all years examined (1970, 1980, 2010): suicide rates were higher for those females who lived in urban than for those who lived in rural regions (but to the end of the observation period, the difference had almost entirely dissipated). A diametrically opposite trend may be observed among males. Very similar results were reported from the nearby Austria regarding the same period [48].

**Table 6:** **Suicide rates (*n*/100,000/year) in different kinds of Hungarian settlements in 1970, 1980, and 2010**

**Type of settlement**	**Gender**	**1970**	**1980**	**2010**
Capital city (Budapest)	Male	41.71	49.55	31.91
	Female	29.12	42.16	11.61
	Total	35.08	45.63	20.89
Towns with county’s rights (a.k.a. urban counties)	Male	43.19	53.74	33.91
	Female	23.00	29.34	10.65
	Total	32.79	41.07	21.44
Towns	Male	50.01	54.13	41.21
	Female	21.99	23.04	10.44
	Total	35.71	38.16	25.18
Villages	Male	54.88	77.57	48.11
	Female	15.19	21.23	9.24
	Total	34.52	48.85	28.26

Source: Hungarian Central Statistical Office, Budapest, Hungary.

#### Seasonal and other temporal components of suicidal behavior

Several epidemiological studies have suggested that the numerical distribution of suicide cases is uneven during the calendar year [49]. According to highly replicated results, there is a peak in the number of suicide cases in the spring and early summer (in some studies, a smaller peak in the autumn for females), and a trough in the winter. In general, violent suicide cases (typical for males) are mainly responsible for the seasonal inequality of suicide rates [49]. Studies show that this type of seasonality is mainly the consequence of the seasonal incidence of depression-related suicides [50,51]. The majority of longitudinal investigations provided some evidence that seasonality in suicidal behavior has decreased in the latest decades [23,52,53], but some studies from other countries have found either increasing or unchanging trends in suicide seasonality [49]. Some studies suggest that the decreasing seasonality of suicides could be a good marker of the lowering rate of depression-related suicides in the population particularly among males [23,53,54]. The Hungarian data are in line with the abovementioned results from other countries. Accordingly, the first Hungarian data set in which the spring peak for suicide was demonstrated was derived from the 1930s (seasonal pattern was similar for both sexes) [55]. Analyses using data from later years (1980–1999 in one study, 1970–2000 in another study, and 1998–2006 in a third study) have also confirmed a peak in the number of suicides in the spring-summer period and a trough in the autumn-winter period [23,56,57]. Only one of these studies investigated genders separately, and this one did not find the abovementioned, autumn peak for females [57]. All three studies reported that seasonal fluctuation of suicide was decreasing during the periods examined [23,56,57]. The one study which investigated seasonal fluctuation of suicide by age found that the decrease pertains only to the young cohorts, while Sebestyén et al. described that the decrease in suicide seasonality is mainly the consequence of the significant decrease among males [23,57]. Tables 7, 8, and 9 present the seasonal distribution of suicide cases in the last decades in the Hungarian total, male, and female populations.

**Table 7:** The proportions (%) of suicide cases by seasons in the Hungarian total population

**Year**	**Spring**	**Summer**	**Autumn**	**Winter**
1980	28.6%	28.8%	23.6%	19.0%
1985	27.1%	30.0%	24.0%	18.9%
1990	28.6%	26.1%	22.3%	22.9%
1995	28.1%	29.0%	21.6%	21.3%
2000	27.2%	29.5%	22.7%	20.6%
2005	28.7%	27.4%	24.2%	19.7%
2010	27.8%	28.3%	25.4%	18.5%

**Table 8:** The proportions (%) of suicide cases by seasons in Hungarian males

**Year**	**Spring**	**Summer**	**Autumn**	**Winter**
1980	29.0%	29.3%	23.2%	18.5%
1985	27.0%	30.2%	24.2%	18.6%
1990	28.1%	26.6%	22.0%	23.3%
1995	28.0%	29.1%	21.6%	21.3%
2000	26.9%	30.1%	22.0%	21.0%
2005	28.9%	27.3%	23.6%	20.2%
2010	26.7%	28.6%	25.2%	19.4%

**Table 9:** The proportions (%) of suicide cases by seasons in Hungarian females

**Year**	**Spring**	**Summer**	**Autumn**	**Winter**
1980	27.7%	27.6%	24.8%	19.9%
1985	27.2%	29.3%	23.6%	19.9%
1990	30.2%	24.9%	23.0%	21.9%
1995	28.5%	28.7%	21.4%	21.3%
2000	28.4%	27.5%	24.7%	19.4%
2005	28.0%	27.8%	26.5%	17.7%
2010	31.8%	26.9%	26.0%	15.3%

Source: Hungarian Central Statistical Office, Budapest, Hungary.

Another highly confirmed and noteworthy result regarding temporal variations of suicide is that more subjects commit suicide in the first days of the week than in the weekend [58,59]. In Hungary, between 1970 and 2002, the average number of suicides peaked on Monday for both sexes and was lowest on Saturday (for males) and Sunday (for females) [59].

### Decreasing suicide mortality in Hungary: what is beyond the figures?

Although the traditionally high suicide rate of Hungary is the second highest in the European community and the fifth to sixth highest in Europe, characteristics of suicidal behavior (gender, age, and urban–rural distribution, method of suicide, marital status, seasonality, rate of psychiatric morbidity) are very similar to those reported from other countries.

In spite of the fact that unemployment and alcohol consumption are well-accepted suicide risk factors [6,7,24,60], these two indices do not show a strong positive correlation with suicide rate in Hungary between 1992 and 2010. However, a significant positive correlation has been found between tobacco consumption and national suicide rate between 1985 and 2008 [8], which may reflect that, as demonstrated also by our studies in Hungary, patients with mood disorders smoke much more frequently than members of the general population [61] and smoking is a suicide risk factor [6,62]. Moreover, smokers are significantly more impulsive than nonsmokers [63,64], and it is well demonstrated that impulsive-aggressive personality features are powerful predictors of suicidal behavior [65,66].

Looking at the problem of suicide from the side of a given individual, there is no doubt that suicidal behavior is the result of the complex interplay between macro-social and personal suicide risk factors, the most powerful of them is current major depression [3-5]. In agreement with international findings, several studies demonstrated the important role of depression in suicidal behavior in Hungary, as we will discuss below. This is particularly important from a practical point of view, as depression is one of most easily amendable suicide risk factor.

Investigating the regional distribution of recognized and treated depressions and suicide rates in 20 regions of Hungary in 1985, 1986, and 1987, the suicide rate showed a significant negative correlation with the rate of treated depressions in each of the 3 years: the higher was the rate of treated depressions and the lower was the suicide rate in the given region. It is also important to note that no such relationship was found regarding treated schizophrenic cases [42].

In a psychological autopsy study conducted more than 25 years ago in Budapest, we have found that 63% of 200 consecutive suicide victims had current depressive disorders (almost half of them had bipolar depression), 9% schizophrenia, and 8% alcoholism [14,67]. More than half of the depressed suicide victims had medical contact during their last depressive episode, but less than 20% of them received antidepressants and/or mood stabilizers [14]. In a most recent case, control psychological autopsy study in 194 suicide victims and 194 controls in Budapest [6] we also found that 60% of victims (and 11% of controls) had current affective disorder, 26% of victims (and 38% of controls) had medical contact and 18% of suicides (and 8% of controls) took antidepressants in the last 4 weeks before the suicide or before the interview. This study also identified a number of societal factors that may be important determinants of the suicide risk in individuals. It has been found that lifetime history of psychiatric illness, such as separated/divorced/widowed marital status, lower educational level, unemployment, or long-term sick/disabled status, adverse life events within the previous 3 months, alcoholism, and current cigarette smoking, was significantly more common among suicide victims, while being responsible for a child less than 18 years of age and practicing a religion was significantly less frequent among the victims than among the controls [6].

Two independent studies on nonviolent suicide attempters (drug overdose or poisoning) in Budapest showed that 69%–87% of the attempters had a current major depressive episode (in many cases with comorbid anxiety and/or substance-use disorders), and unemployment, living alone, and economically inactive status were overrepresented among them [30,68]. The strong relationship between suicide attempts and agitated/mixed depression has been also found both in population-based epidemiological [15] and clinical samples [69].

While the suicide rate of Hungary showed a steady (46%) decline between 1983 and 2006, most of the ‘post-communist’ countries (e.g., the Baltic States) exhibited decrease in their suicide mortality only from the mid 1990s, several years after the big political/economic changes started around 1990 (the suicide rate of some other ‘post-communist’ countries, e.g., Poland and Romania, has reached its zenith even later (in 2005 and 2000, respectively)) [2]. On the other hand, however, the greatest decline in national suicide rate on the world (more than 65%) between the mid 1980s and 2010 were detected in Denmark, that is not a typical ‘post-communist’ country [2]. This shows that political/economic change is probably not the main factor behind this favorable trend. At the same time, between 1983 and 2006, the prescription of antidepressants increased by tenfold. The negative correlation between antidepressant prescription and national suicide rate in Hungary between 1985 and 2011 is well demonstrated in several previously published papers showing that better recognition and more widespread treatment of depressive disorders, as reflected in the increasing antidepressant utilization, seems to be one of the main contributing factors in the markedly declined suicide rate of Hungary in the last 3 decades [8,21,23,29,34,70]. Similarly, a statistically significant correlation between increasing antidepressant utilization and decreasing national suicide rates have been reported recently from several countries [35,71], including Sweden, Denmark, Finland, Norway [3,72,73], the USA [74], Japan [75] and, as mentioned above, Hungary [8,21,23,29,34,70]. Although ecological association does not mean causality, considering that

1) there is a strong relationship between untreated major depression and suicide [5,76,77];

2) the appropriate acute and long-term treatment of patients with major depressive and bipolar disorders markedly reduces the suicide mortality even in this high-risk patient-population [5,76,77] and initially suicidal depressives become nonsuicidal with antidepressant treatment [5,78]; and

3) the annual prevalence of major depressive episode in the population is around 6%–8% [18,79].

It is logical to assume that more widespread treatment of depression is one of the main causes of declining suicide rates in countries where antidepressant utilization recently increased markedly. On the other hand, however, as national suicide rates are affected by many known (unemployment, divorce rate, alcohol consumption, living standard, etc.) and unknown factors [80,81], the isolation of the result of better treatment of depression in declining suicide rates is not easy.

The increase in antidepressant utilization, as reflected in antidepressant prescriptions, is only a proxy marker of greater access of patients to appropriate care, and higher population density of doctors in general [82,83] and psychiatrists and psychotherapists in particular [21,22,83] are negatively associated with national and regional suicide rates. It is likely that many patients receiving antidepressants also receive a prescription of lithium and other mood stabilizers as well as they receive more frequently supportive or specific psychotherapy for depression. Between 1982 and 2000, the number of psychiatrists in Hungary increased from 550 to 850, as well as the number of outpatient psychiatric departments (from 95 to 139), and the number of S.O.S. telephone services (from 5 to 28) [70]. It should be also noted that between 1990 and 2010, the number of telephones (the best mean for rapid communication even in the case of suicidal crisis) increased by fivefold in Hungary, and recently, the number of ordinary and mobile phones is over 11 million while the population of the country is 10 million. Although it is not possible to measure, it is very likely that the new democratic political system since 1990 (including freedom of religion and several newly founded civil organizations) plays also important role in this favorable process. Therefore, the decrease of suicide rates could reflect a general improvement in mental health care rather than being caused by increasing antidepressant sales alone. The robust increase of antidepressant prescription in Hungary remains the only consistent correlate of declining national suicide rate of Hungary in the last 25–30 years [8,21,23,34,70], indicating that better recognition and treatment of depression is one, but not the only, important contributor to this favorable change. On the other hand, however, recently, we have suggested that increasing unemployment rate after 2005 might be one of the contributing factors that accounted for the disappearance of the strong decreasing trend from the Hungarian suicide rate that stabilized around 24/100,000 between 2006 and 2011 [25]. The exact causes, the role of possible contributory factors as well as the relationship between them remain to be elucidated, yet it seems increasingly obvious that patterns and trends in suicide rates in Hungary are determined by a delicate interplay between various genetic, psychiatric, cultural, economic, political, social, and treatment-related factors specific for Hungary.

In spite of the great decline in the suicide rates of Hungary in recent decades most likely resulting from advancements in Hungarian healthcare well indicating the possibilities for suicide prevention, there is, unfortunately, no centrally directed, government-organized suicide prevention program in Hungary. However, in the last 20–25 years the importance of psychiatric disorders (particularly depression) in suicide and suicide prevention receives more and more attention in the training of medical students, residents, psychiatrists, and general practitioners. These regular trainings are organized by the four medical universities (Budapest, Pécs, Szeged, Debrecen), the Hungarian Psychiatric Association, the Association of Hungarian Neuropsychopharmacologists, and also by pharmaceutical companies. In spite of the fact that the decline of national suicide mortality in Hungary in the last 25 years (46%) is among the greatest decreases in the world, the suicide rate of Hungary is still very high (in 2011, 24.3/100,000), so much remains to be done.

In agreement with the findings of the Swedish Gotland Study [84], our community-intervention suicide preventive project in the Kiskunhalas region of Hungary (68,000 inhabitants) between 2000 and 2005 showed that education of doctors, other healthcare professionals, and gatekeepers, as well as the public, is an effective method of reducing suicide mortality [85]. The Hungarian Depression Recognition and Suicide Prevention Program in Szolnok between 2004 and 2006 (as a part of the European Alliance Against Depression program) directed by Maria Kopp also supported the key role of general practitioners and other gatekeepers (psychiatrists, psychologists, telephone help service providers, pharmacists, teachers, pastors, police officers, family nurses, geriatric care providers, etc.) in suicide prevention [86].

Screening and also medical care for those at increased risk of suicide should be extended from psychiatric practices to all specialities of healthcare, especially to primary care, and primary care providers should not only be qualified to screen for depression and suicide risk, but should also provide subsequent care for previous suicide attempters since previous attempts are among the major risk factors for suicide. Also, in primary care practices, family suicide events, another important risk factor, should also be screened for [87]. General practitioners are in the best position to detect if multiple important risk factors of the suicide constellation are present in case of their patients and could provide referral to mental health care or counselling services.

As psychosocial and community factors also play an important role in suicidal behavior, it is not only health care workers who are responsible for its prevention. Improving well-being and quality of life of people in general (including decreasing unemployment and providing more support for health and social services), restricting lethal suicide methods (e.g., reducing domestic and car exhaust gas toxicity and introducing stricter laws on gun control), and initiating more restricitve alcohol and smoking policies, which may also reduce suicide mortality [4,9,88,89], exceed the limits and jurisdiction of health and social care and are rather the leaders’ competence and responsibility at any level of the society. Individual programs developed and implemented in each level of the society, however, need to be coordinated by a unified, government-level central suicide prevention plan. A central prevention plan should appoint possible targets of prediction, prevention, and intervention, at multiple levels and building on multiple groups within the society. The suicide prevention plan should include guidelines for data collection concerning suicide and its risk factors; outline of necessary changes in medical training; training for workers in the social field; and also training for church associated persons as well as teachers and policemen; enhancing scientific research in related fields, tackling ethical, moral, and legal issues related to suicide; and changing legislation if necessary, designing and launching public campaigns, planning enlightenment campaigns, and creating possibilities for interventions in schools. Coordinators at all levels should also be appointed. However, for such a plan to be realized, first awareness concerning the burning issue of suicide in Hungary should be raised not only for the population but also for politicians, legislators, and health/social care decision makers.

## Conclusion

In our review, we tried to collect the current knowledge and data on the main topics of the Hungarian suicide scene and also on the pilot projects which were conducted in order to better prevention of suicide cases. Our primary aim with this review was to draw attention to the severity of suicide scene in Hungary, a problem which has however shown an improving trend in the last decades but still poses a serious burden to the society.

We also hope that these raw data, and our inferences and proposals based on them, will be taken into account by the political and academic decision makers when they will consider the necessity of the launching of a nationwide prevention program and the concommitant scientific research on the background mechanisms as well.

We are, of course, unable to prevent all suicides. Nevertheless, the already existing theoretical knowledge and the available treatment and preventive strategies are sufficient to prevent many, probably the majority, of them.

## Competing interests

The authors declare that they have no competing interests.

## Authors’ contributions

ZR and PD conceived the main idea of this review. BK gathered raw data. XG conducted literature search. All authors participated in preparing the manuscript. All authors read and approved the final manuscript.
